# Supplementary material for: Climatic variation in Africa and Europe has combined effects on timing of spring migration in a long-distance migrant Willow Warbler Phylloscopus trochilus
Source: PeerJ. 2020 Mar 17;8:e8770. doi: 10.7717/peerj.8770 (PMC7083157; doi:10.7717/peerj.8770)
Supplement: Supplemental Information 1 [file peerj-08-8770-s001.pdf]

Climatic variation in Africa and Europe has combined effects on timing of spring migration in a long-distance migrant bird: a case study on Willow Warbler *Phylloscopus trochilus*

Supplementary Information
Figures S1–S4 and Tables S1–S9

Magdalena Remisiewicz[1,2], Les G. Underhill[2,3]

[1] Bird Migration Research Station, Faculty of Biology, University of Gdańsk, Wita Stwosza 59, 80-308, Gdańsk, Poland; http://orcid.org/0000-0002-3613-5738

[2] Animal Demography Unit, Department of Biological Sciences, University of Cape Town, Rondebosch, 7701, South Africa; http://orcid.org/0000-0002-8758-1527

[3] Biodiversity and Development Institute, Unit 4, Gunner's Park, Gunner's Circle, 7475, South Africa

Corresponding Author:
Magdalena Remisiewicz

Email address: magdalena.remisiewicz@ug.edu.pl

Figure S1. Cumulative count of Willow Warblers in each spring and the multi-year average cumulative count in 1982–2017. The area between the two curves reflects the Annual Anomaly for a season: the area on the left of the many-year curve, which represents the advance of the annual curve in relation to the many-year average, was subtracted from the area on the right of the of the many-year curve, which represents the delay, to obtain the overall value of AA.

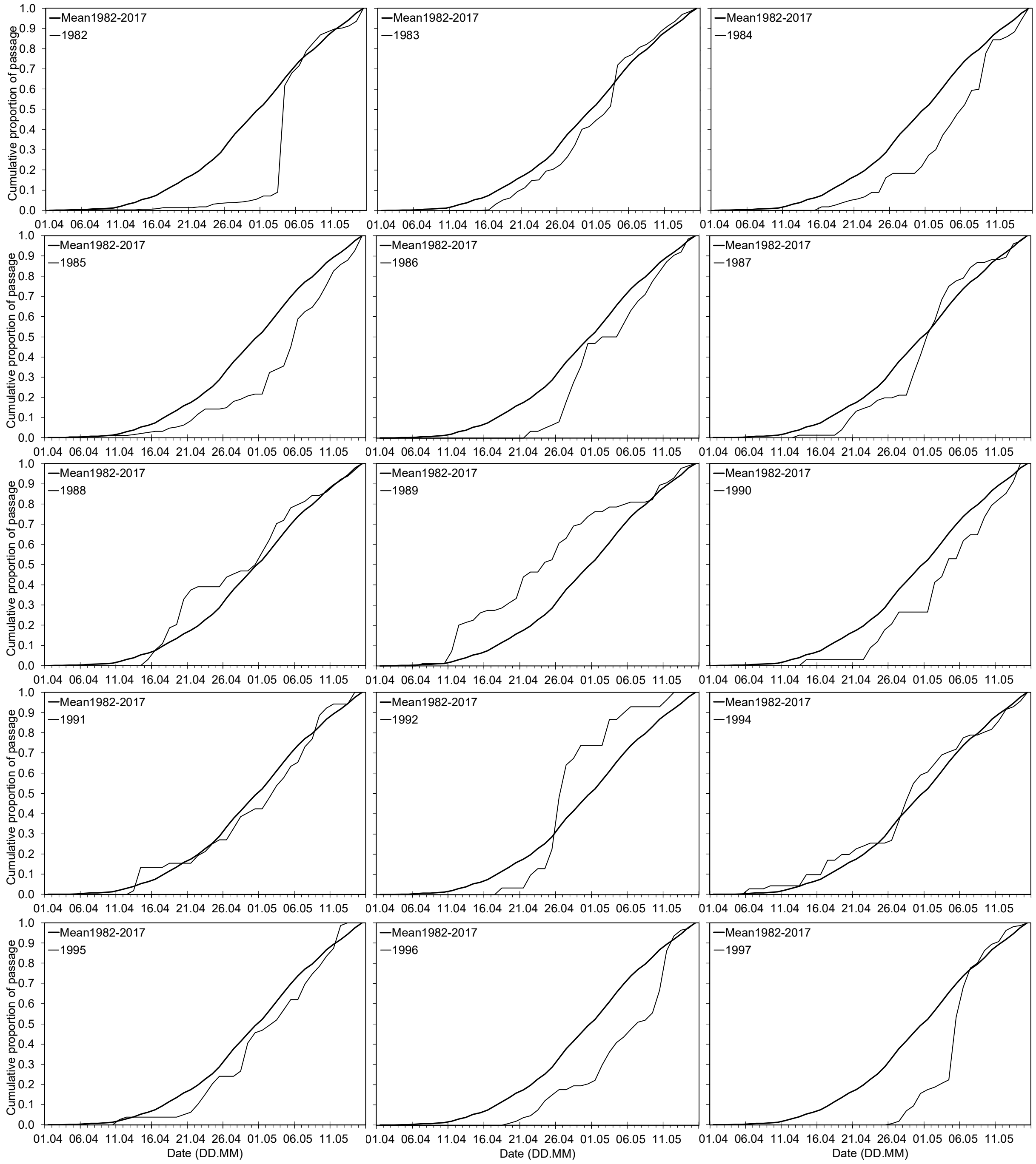

Figure S1 continued.

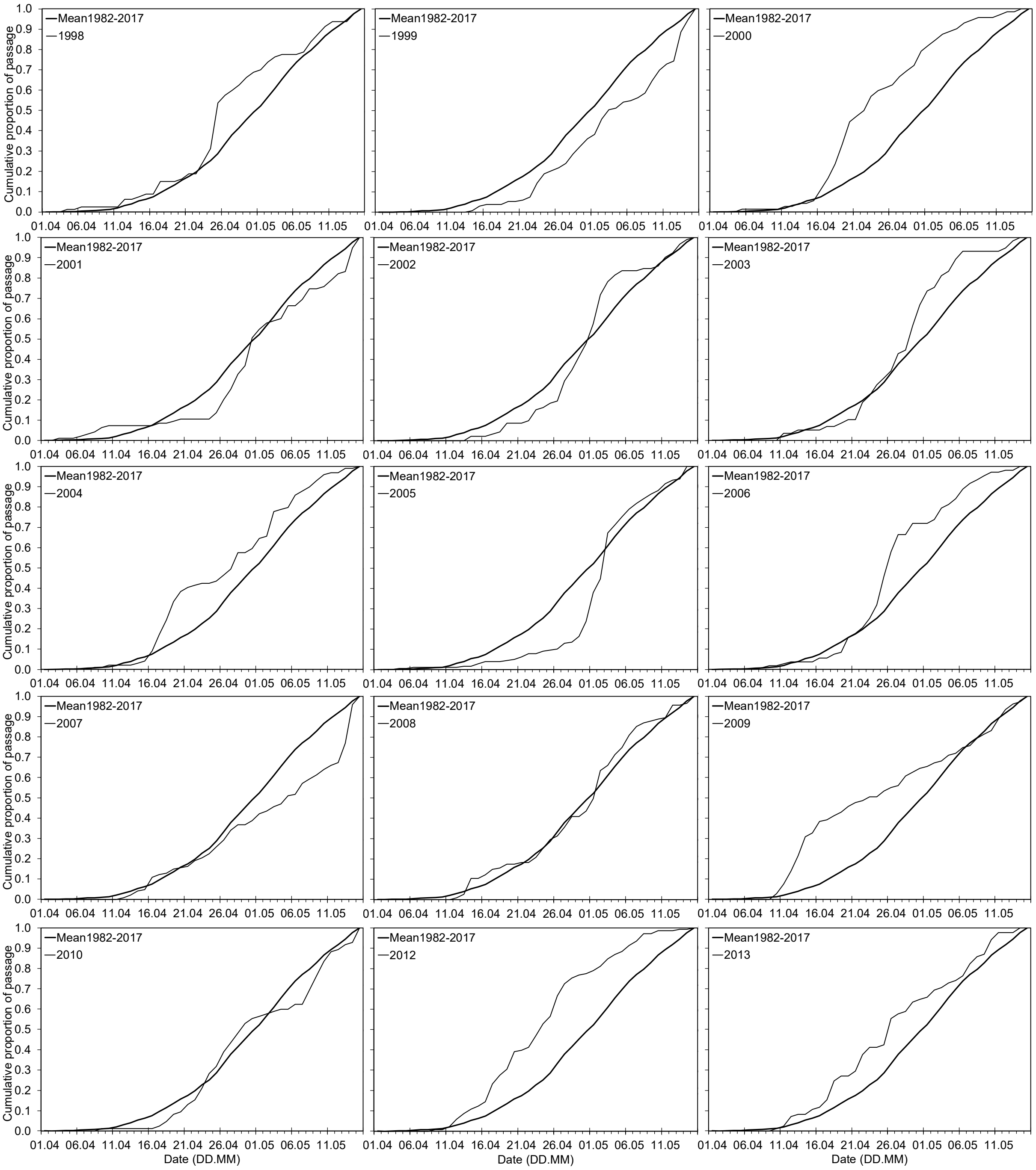

Figure S1 continued.

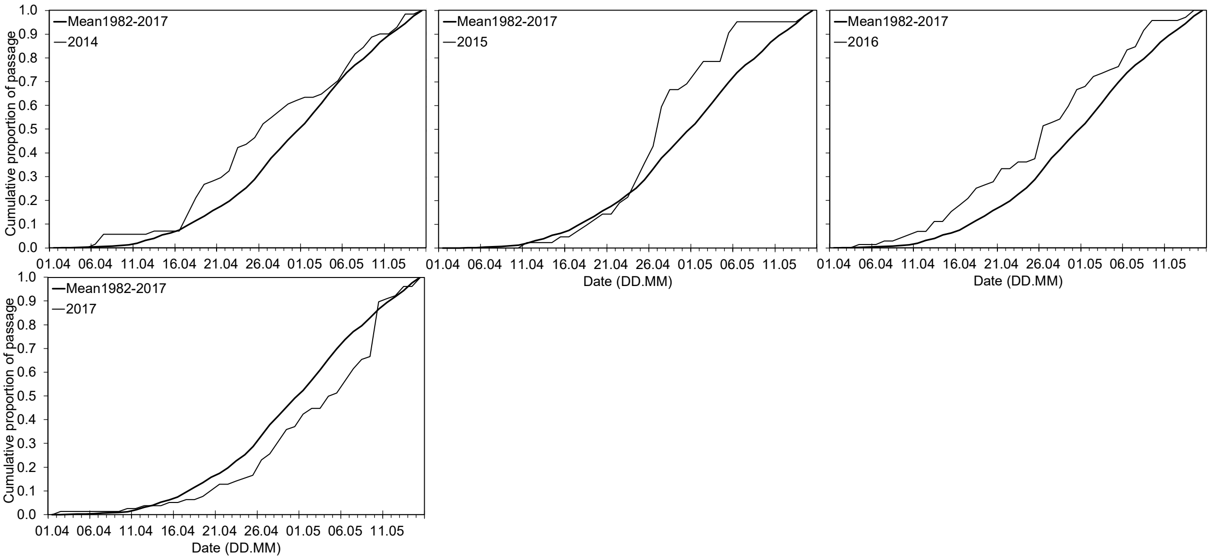

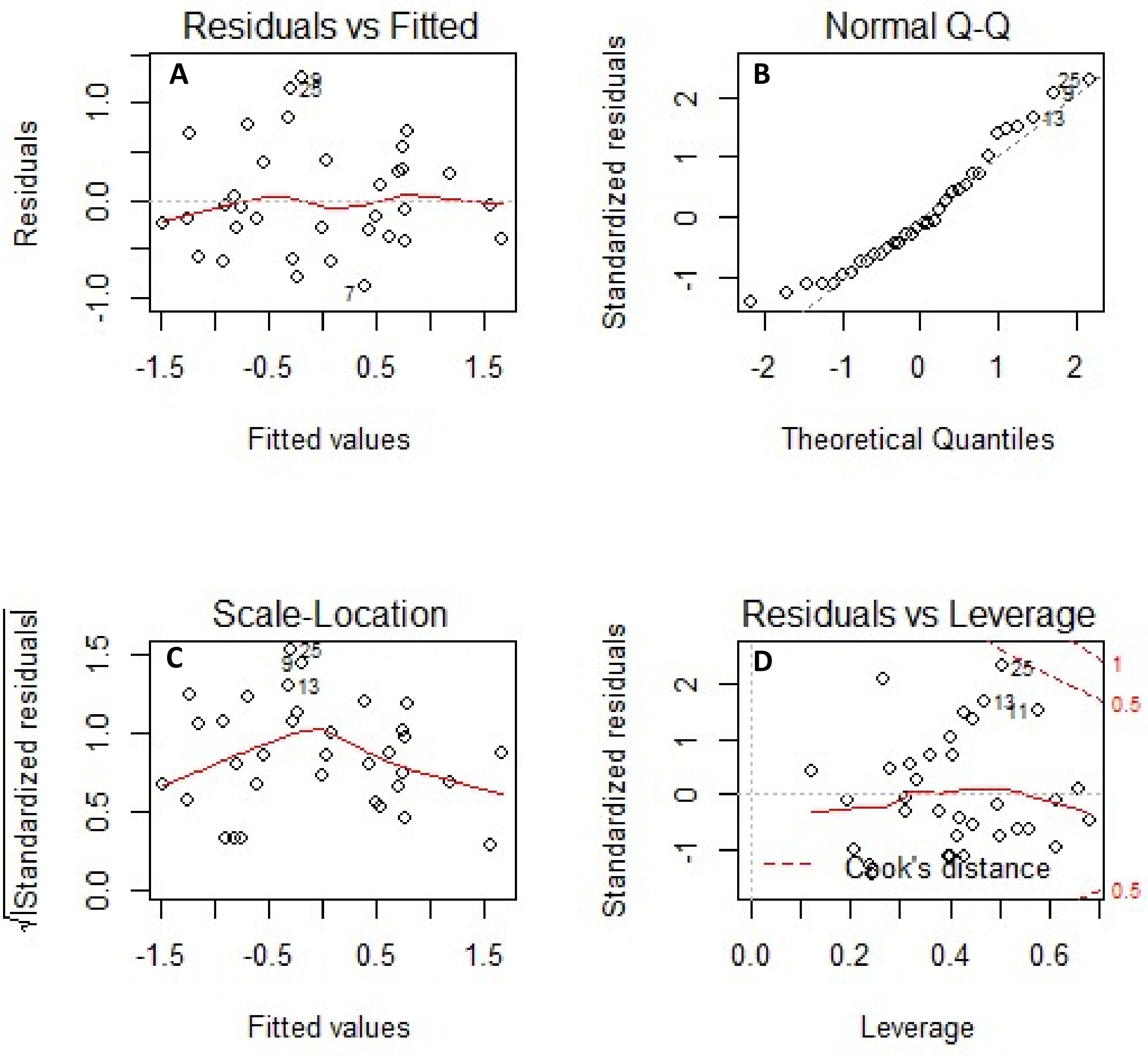

Figure S2. Model diagnostics for the full model with 13 climate variables and the Year (Table S5). The plots of residuals follow Crawley (2013).

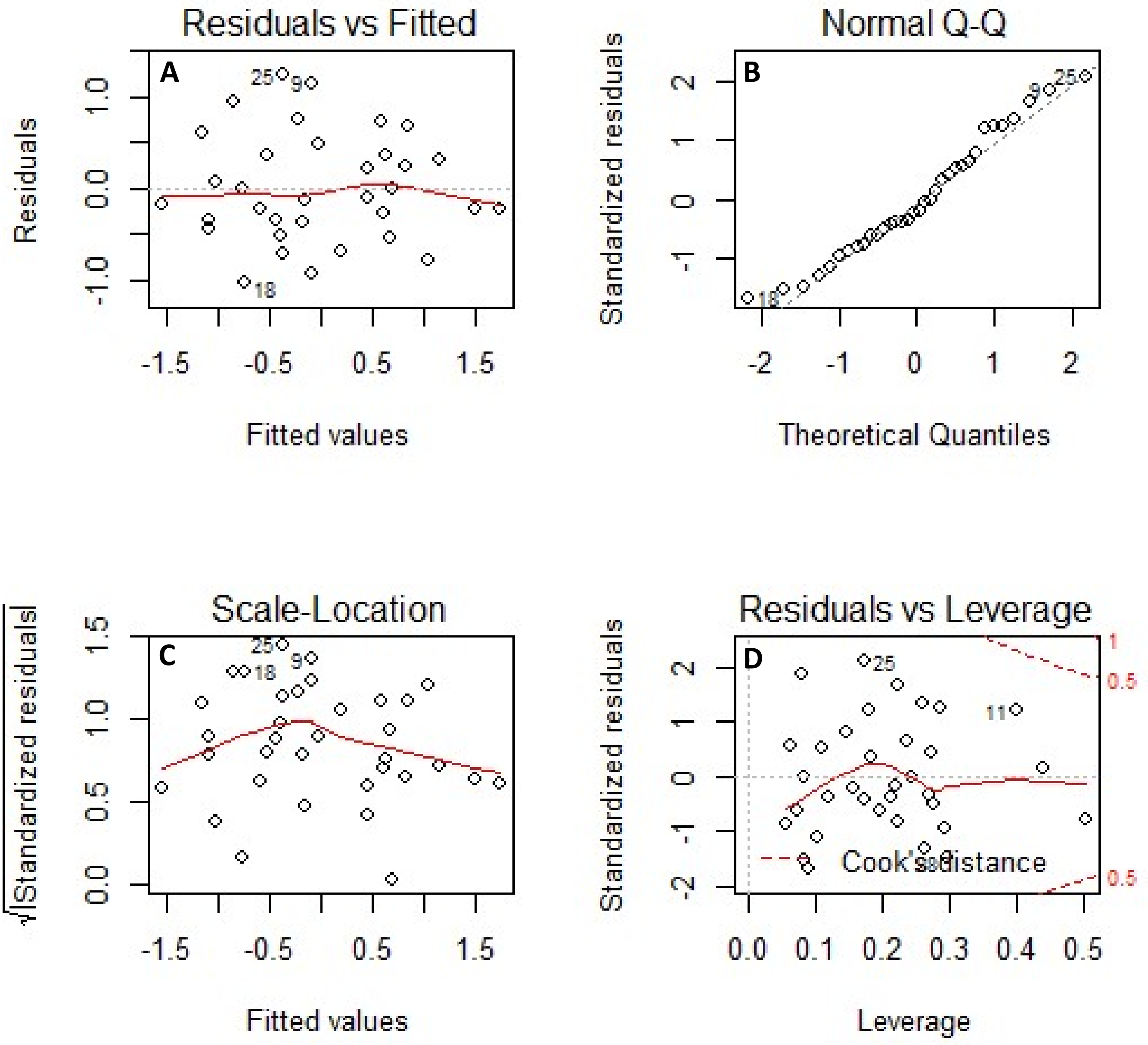

Figure S3. Model diagnostics for the best-fitted model (Table 2). The plots of residuals follow Crawley (2013).

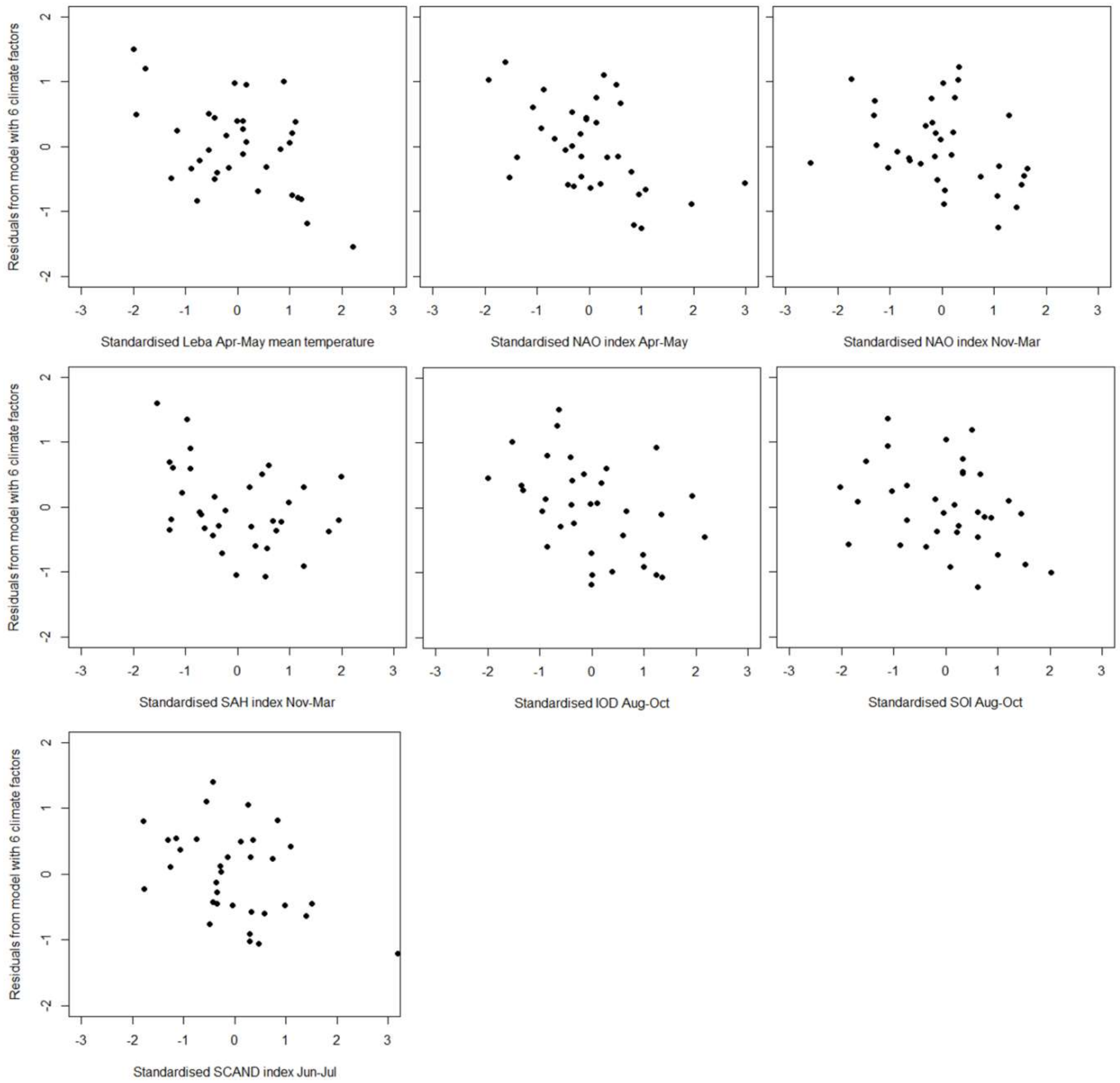

Figure S4. Residuals computed from a model with six variables plotted against each of the remaining single variables from the best-fitted model with seven climate variables that explained variation in the Annual Anomaly (AA) at Bukowo, Poland, in 1982–2017 (Table 2). The scattered points indicate that inclusion of each variable (marked at X-axis) was not driven by the data for a single year but explained an additional proportion of the variation in AA.

Table S1. Counts of Willow Warblers in each year of the study at Bukowo. N = numbers of Willow Warblers aged as “full grown” during spring migration (1 April–15 May) used to calculate the Annual Anomaly (AA) for each spring; “–” = years excluded from analyses because fewer than 30 Willow Warblers were caught.

Year	N	AA
1981	–	–
1982	1029	5.14
1983	504	1.27
1984	181	4.54
1985	189	4.41
1986	64	3.47
1987	78	1.19
1988	65	-1.65
1989	84	-5.28
1990	34	3.70
1991	52	0.48
1992	31	-1.87
1993	22	–
1994	73	-0.97
1995	79	1.86
1996	108	5.25
1997	165	5.30
1998	82	-2.69
1999	141	3.72
2000	74	-6.02
2001	99	1.56
2002	92	0.35
2003	52	-1.87
2004	119	-3.49
2005	178	2.34
2006	108	-3.09
2007	151	2.96
2008	117	-0.56
2009	111	-4.98
2010	86	0.90
2011	–	–
2012	139	-5.94
2013	89	-3.25
2014	81	-2.84
2015	47	-2.63
2016	93	-3.73
2017	89	2.41

Table S2. Pearson's correlation coefficients between the year (1982–2017) and the climate indices used in the study. Significant correlations ($P < 0.05$) marked in bold face. VIF – variance inflation factors in the full model with all variables included. Abbreviations of climate variables: LEB Apr–May = Apr–May mean of the daily means of temperatures in Łeba; NAOI Apr–May = Apr–May mean of the Northern Atlantic Oscillation Index; SCAND Apr–May = Apr–May mean of the Scandinavian Pattern Index; NAOI Nov–Mar = Nov–Mar mean of the Northern Atlantic Oscillation Index; SAH Nov–Mar = Nov–Mar mean of the Sahel Precipitation Anomaly; IOD Nov–Mar = Nov–Mar mean of the Indian Ocean Dipole; SOI Nov–Mar = Nov–Mar mean of the Southern Oscillation Index; NAOI Aug–Oct = Aug–Oct mean of the Northern Atlantic Oscillation Index; SAH Aug–Oct = Aug–Oct mean of the Sahel Precipitation Anomaly; IOD Aug–Oct = Aug–Oct mean of the Indian Ocean Dipole; SOI Aug–Oct = Aug–Oct mean of the Southern Oscillation Index; NAOI Jun–Jul = Jun–Jul mean of the Northern Atlantic Oscillation Index; SCAND Jun–Jul = Jun–Jul mean of the Scandinavian Index. April–May precipitation in Łeba was not included in the full model, hence no VIF for this variable.

Variable	Year	LEB Apr–May	NAOI Apr–May	SCAND Apr–May	NAOI Nov–Mar	SAH Nov–Mar	IOD Nov–Mar	SOI Nov–Mar	NAOI Aug–Oct	SAH Aug–Oct	IOD Aug–Oct	SOI Aug–Oct	NAOI Jun–Jul	VIF
Year														1.20
LEB T Apr–May	**0.48**													1.46
NAOI Apr–May	–0.14	–0.09												1.31
SCAN Apr–May	–0.22	–0.08	–0.02											1.96
NAOI Nov–Mar	0.05	0.02	0.17	–0.10										1.54
SAH Nov–Mar	0.25	0.07	–0.01	0.00	0.07									1.21
IOD Nov–Mar	**0.37**	0.06	–0.27	–0.01	–0.01	–0.09								3.51
SOI Nov–Mar	0.19	–0.02	–0.02	–0.20	–0.06	0.05	–0.11							4.77
NAOI Aug–Oct	–0.27	–0.31	–0.05	0.28	–0.12	0.00	**–0.37**	–0.09						1.97
SAH Aug–Oct	**0.52**	0.28	0.02	–0.15	0.27	0.11	**0.39**	0.11	–0.29					1.88
IOD Aug–Oct	0.23	0.07	–0.18	0.27	0.19	–0.08	**0.60**	**–0.41**	–0.21	0.24				3.21
SOI Aug–Oct	0.05	–0.16	0.15	–0.18	–0.22	–0.09	–0.33	**0.82**	**0.11**	**–0.02**	**–0.61**			7.09
NAOI Jun–Jul	**–0.40**	0.16	0.14	**0.37**	–0.11	0.11	**–0.46**	–0.10	0.02	–0.13	–0.31	–0.03		2.35
SCAND Jun–Jul	–0.02	**–0.39**	–0.09	0.16	0.00	–0.22	**0.35**	0.06	0.26	–0.17	0.23	0.08	**–0.38**	2.09
LEB precipitation Apr–May	0.12	0.28	–0.21	0.13	**–0.44**	–0.03	0.26	–0.09	–0.02	0.10	–0.10	–0.00	0.22	–

Table S3. Summary statistics for linear regressions over the year in 1982–2017 for the climate variables used in the study. June–July and August–October means are averages in 1981–2016 preceding Willow Warblers' spring migrations in 1982–2017. Values averaged for the given range of months and used as raw (non-standardised) data. Abbreviations of variables as in Table S2. $P < 0.05$ marked in bold face.

Parameter	*ß* slope	SE	R^2	t_{32}	*P*
**LEB Apr–May**	**0.0406**	0.0132	0.23	3.07	**0.0044**
NAOI Apr–May	-0.0099	0.0123	0.02	-0.80	0.4272
SCAN Apr–May	0.0028	0.0104	0.002	0.27	0.7902
NAOI Nov–Mar	0.1403	0.0962	0.06	1.46	0.1543
SAH Nov–Mar	0.0177	0.0165	0.03	1.07	0.2907
**IOD Nov–Mar**	**0.0066**	0.0030	0.13	2.22	**0.0338**
SOI Nov–Mar	0.0176	0.0088	0.11	1.99	0.0548
NAOI Aug–Oct	-0.0143	0.0090	0.07	-1.60	0.1204
**SAH Aug–Oct**	**8.4100**	2.4360	0.27	3.45	**0.0016**
IOD Aug–Oct	0.0034	0.0134	0.002	0.26	0.8004
SOI Aug–Oct	0.0083	0.0062	0.05	1.35	0.1871
**NAOI Jun–Jul**	**-0.0310**	0.0127	0.16	-2.44	**0.0204**
SCAND Jun–Jul	-0.0139	0.0165	0.02	-0.84	0.4065

Table S4. Summary statistics for linear regressions over the year for the metrics of spring migration phenology of Willow Warblers in 1982–2017. 5%–95% = subsequent percentiles of spring migrants, AA = the Annual Anomaly, Duration 10%–90% = the difference between the dates when 10% and 90% of migrants passed through Bukowo, Mean = many-year mean date, "–" = mean date not applicable, *ß* slope = regression coefficient; SE = its Standard Error; R^2 – determination coefficient; *t, P* = results of *t*-test, $p < 0.05$ marked in bold face, 36 x ß = Estimated change in days of timing over 1982–2017, negative values reflect advance of migration.

Parameter	Mean	SD	ß slope	SE	R^2	t_{32}	p	36 x ß (days)
5%	17 Apr	5.03	-0.27	0.07	0.32	-3.92	**0.0004**	-9.6
10%	20 Apr	5.03	-0.22	0.08	0.22	-2.97	**0.0057**	-7.9
25%	25 Apr	5.14	-0.24	0.08	0.22	-3.03	**0.0049**	-8.3
50% (median)	30 Apr	3.96	-0.17	0.06	0.20	-2.80	**0.0087**	-6.1
75%	6 May	3.28	-0.06	0.06	0.03	-1.04	0.3061	-3.2
90%	11 May	2.59	-0.10	0.04	0.15	-2.40	**0.0222**	-5.2
95%	12 May	2.49	-0.06	0.04	0.06	-1.46	0.1543	-3.2
Duration 10%–90%	–	–	0.17	0.08	0.12	2.11	**0.0428**	6.2
AA	–	–	-0.15	0.05	0.21	8.68	**0.0059**	-5.4

Table S5. Relationship between the Annual Anomaly for the timing of Willow Warblers' spring migration at Bukowo, Poland, in 1982–2017, and the 13 climate variables and the year, in full model. Full model statistics: $F_{14,20} = 3.329$, $P = 0.0072$, $AdjR^2 = 49.0\%$ Estimate – coefficients from multiple regression, SE – standard error of the estimates, *t, P* – *t*-test and significance of each estimate, VIF – variance inflation factor, R^2 – partial R^2 coefficients. $P < 0.05$ marked in bold face. Abbreviations of variables as in Table S2.

Explanatory variable	Estimate	SE	*t*	*P*	VIF	R^2
YearN	–0.001	0.006	–0.19	0.852	1.20	0.0018
**LEB Apr–May**	–0.397	0.148	–2.69	**0.014**	1.46	0.2652
**NAO Apr–May**	–0.410	0.140	–2.93	**0.008**	1.31	0.2997
SCAND Apr–May	0.001	0.172	0.01	0.995	1.96	0.0000
NAO Nov–Mar	–0.219	0.152	–1.44	0.166	1.54	0.0936
SAH Nov–Mar	–0.215	0.135	–1.59	0.127	1.21	0.1125
IOD Nov–Mar	–0.126	0.230	–0.55	0.590	3.51	0.0148
SOI Nov–Mar	–0.036	0.268	–0.13	0.895	4.77	0.0009
NAO Aug–Oct	–0.135	0.172	–0.78	0.443	1.97	0.0297
SAH Aug–Oct	–0.117	0.168	–0.70	0.495	1.88	0.0236
**IOD Aug–Oct**	–0.457	0.220	–2.08	**0.051**	3.21	0.1776
SOI Aug–Oct	–0.306	0.326	–0.94	0.360	7.09	0.0421
NAO Jun–Jul	0.068	0.188	0.36	0.722	2.35	0.0064
SCAND Jun–Jul	–0.333	0.177	–1.88	0.075	2.09	0.1502

Table S6. Comparison of regression models of Annual Anomaly (AA) against each climate variable used as a linear term and as a quadratic term. m1 – linear model, m2 – quadratic model. The models were ranked by corrected Akaike's Information Criteria for small samples size (AICc), *k* is the number of estimated parameters in the model, ΔAICc gives the difference in AICc from the model with lowest AICc. Negative ΔAICc indicate that the quadratic model was better than the linear model, ΔAICc < –2 indicate a considerably better fit of the quadratic model. Abbreviations of variables as in Table S2.

Climate variable	Models	*k*	AICc	ΔAICc
LEB Apr–May	m1	2	98.12	
	m2	3	100.43	2.32
NAOI Apr–May	m1	2	96.84	
	m2	3	99.24	2.39
SCAND Apr–May	m1	2	99.84	
	m2	3	99.63	–0.21
NAOI Nov–Mar	m1	2	95.49	
	m2	3	96.11	0.62
SAH Nov–Mar	m1	2	99.46	
	m2	3	100.70	1.24
IOD Nov–Mar	m1	2	95.89	
	m2	3	97.16	1.28
SOI Nov–Mar	m1	2	99.58	
	m2	3	100.28	0.71
NAOI Aug–Oct	m1	2	99.57	
	m2	3	99.50	–0.06
SAH Aug–Oct	m1	2	94.07	
	m2	3	96.07	2.01
IOD Aug–Oct	m1	2	93.64	
	m2	3	92.30	–1.34
SOI Aug–Oct	m1	2	99.89	
	m2	3	99.57	–0.32
NAO Jun–Jul	m1	2	96.66	
	m2	3	94.17	–2.49
SCAND Jun–Jul	m1	2	96.56	
	m2	3	98.85	2.29

Table S7. Model selection procedure by "all subsets" selection, according to AICc, from the full model (Table S5). The models describe relationship between the Annual Anomaly (AA) for the timing of Willow Warblers' spring migration at Bukowo, Poland, in 1982–2017, and the 13 climate variables and the year (Table 1). The table presents all models with ΔAICc < 2. The models were ranked by corrected Akaike's Information Criteria for small samples size (AICc), *k* is the number of estimated parameters in the model, ΔAICc gives the difference in AICc from the model with lowest AICc, w_i is the Akaike weight. The best model, discussed in the text, is given in bold face. Model selection conducted using package MuMIn 1.43.6 (Bartoń, 2019) in R 3.4.4 (R Core Team, 2018). Abbreviations of variables as in Table S2.

Model formula	*k*	AICc	ΔAICc	W_i
**AA~IOD Aug-Oct + LEB Apr-May + NAO Apr-May + NAO Nov-Mar + SAH Nov-Mar + SCAN Jun-Jul + SOI Aug-Oct**	**8**	**80.4529**	**0.0000**	**0.1620**
AA~IOD Aug-Oct + LEB Apr-May + NAO Apr-May + SAH Aug-Oct + SCAN Jun-Jul + SOI Nov-Mar	7	81.7704	1.3174	0.0838
AA~IOD Aug-Oct + LEB Apr-May + NAO Apr-May + SAH Aug-Oct + SAH Nov-Mar + SCAN Jun-Jul + SOI Aug-Oct	8	81.7708	1.3179	0.0838
AA~IOD Aug-Oct + LEB Apr-May + NAO Apr-May + SAH Aug-Oct + SCAN Jun-Jul	6	81.7786	1.3257	0.0835
AA~IOD Aug-Oct + LEB Apr-May + NAO Apr-May + SAH Nov-Mar + SCAN Jun-Jul + SOI Aug-Oct	7	81.8355	1.3826	0.0812
AA~IOD Aug-Oct + LEB Apr-May + NAO Apr-May + SAH Aug-Oct + SAH Nov-Mar + SCAN Jun-Jul	7	81.9157	1.4627	0.0780
AA~IOD Aug-Oct + LEB Apr-May + NAO Apr-May + SAH Nov-Mar + SCAN Jun-Jul + SOI Nov-Mar	7	81.9201	1.4672	0.0778
AA~IOD Aug-Oct + LEB Apr-May + NAO Apr-May + SAH Aug-Oct + SAH Nov-Mar + SCAN Jun-Jul + SOI Nov-Mar	8	82.0093	1.5564	0.0744
AA~IOD Aug-Oct + LEB Apr-May + NAO Apr-May + NAO Nov-Mar + SCAN Jun-Jul + SOI Aug-Oct	7	82.0133	1.5603	0.0743
AA~IOD Aug-Oct + LEB Apr-May + NAO Apr-May + NAO Nov-Mar + SAH Aug-Oct + SAH Nov-Mar + SCAN Jun-Jul + SOI Aug-Oct	9	82.1730	1.7200	0.0686
AA~IOD Aug-Oct + LEB Apr-May + NAO Apr-May + SCAN Jun-Jul + SOI Nov-Mar	6	82.1779	1.7249	0.0684
AA~IOD Aug-Oct + LEB Apr-May + NAO Apr-May + NAO Nov-Mar + SCAN Jun-Jul + SOI Nov-Mar	7	82.3013	1.8483	0.0643

Table S8. Summary statistics for linear regressions of the IOD (August–October) over the SOI (August–October) in 1981–2016. The mean values are from August–October in 1981–2016 preceding Willow Warblers' spring migrations in 1982–2017, which was analysed in this study. Both variables standardised. Symbols as in Table S4.

Parameter	*ß* slope	SE	R^2	$t_{1,33}$	*P*
IOD_AUG_OCT	-0.6053	0.1384	0.3669	-4.373	**0.0001**

Table S9. Summary statistics and correlations of AA and dates of percentiles of spring migration phenology of Willow Warblers at Bukowo in 1982–2017. All variables are used as raw data. *r* - Pearson's coefficients for correlations between AA and each remaining variable. Significant correlations ($p < 0.05$) marked in bold face. N= 34 years (1993 and 2011 excluded because of small numbers of birds). Symbols as in Table S4.

Parameter	*r*	Mean	SD
5%	**0.71**	16.6	5.0
10%	**0.78**	19.7	5.1
25%	**0.91**	24.8	5.2
50% (median)	**0.95**	30.1	4.1
75%	**0.79**	35.9	3.5
90%	**0.65**	40.5	2.7
95%	**0.39**	42.1	2.6
Duration 10%–90%	**-0.35**	23.9	5.2
AA	–	0.0	3.4

REFERENCES

Bartoń K. 2019.Package 'MuMIn'. Multi-Model Inference. *Available at https://cran.r-project.org/web/packages/MuMIn/MuMIn.pdf.* (accessed November 20, 2019).

Crawley MJ. 2013. *The R Book.* Chichester, England: John Wiley & Sons.

R Core Team. 2018.R: a language and environment for statistical computing. Available at *https://www.r-project.org*
